# Loneliness Is Associated With Problematic Internet Use but Not With the Frequency of Substance Use: A Czech Cross-Sectional Study

**DOI:** 10.3389/ijph.2023.1606537

**Published:** 2023-11-02

**Authors:** Filip Meckovsky, Jana Furstova, Alice Kosarkova, Zdenek Meier, Peter Tavel, Klara Malinakova

**Affiliations:** ^1^ Olomouc University Social Health Institute, Palacky University Olomouc, Olomouc, Czechia; ^2^ Department of Christian Education, Sts Cyril and Methodius Faculty of Theology, Palacky University Olomouc, Olomouc, Czechia

**Keywords:** loneliness, social isolation, problematic internet use, substance use, COVID-19

## Abstract

**Objectives:** This study aimed to assess the associations between loneliness and the frequency of substance use and problematic Internet use (PIU) in different age groups.

**Methods:** Data were collected in April 2021 from a sample of 1,293 participants with main characteristics close to a nationally representative sample (mean age = 50.1 ± 15.4 years; 56% male). We measured loneliness with the Three-Item Loneliness Scale (TILS), PIU with the General Problematic Internet Use Scale-2 (GPIUS-2) and the frequency of drugs, alcohol, smoking or caffeine consumption. Spearman’s correlation, the t-test, and one-way and multivariate linear regression models were used to analyze the data.

**Results:** In our study, 43.8% of respondents reported moderate to severe levels of loneliness. Loneliness was associated with the severity of PIU [F (3, 1,277) = 15.25, *p* < 0.001], with higher loneliness corresponding to higher PIU. No significant relationship was found between loneliness and drugs, alcohol, smoking or caffeine consumption.

**Conclusion:** Regardless of age, loneliness is associated with PIU but not with the frequency of substance use. Professional help for lonely people should focus on problematic Internet use in all age groups.

## Introduction

Due to its severe health consequences, loneliness is considered one of the major public health threats in the 21st century [1, 2]. It is defined as a subjective socioemotional state of missing quality relationships [3], which involves feelings of not belonging and disconnectedness [4]. As the prevalence of loneliness has rapidly increased due to the COVID-19 pandemic [5, 6] and is placing a burden on both the health and the economic systems, researchers are urging governments to put loneliness among the top health policy priorities [7, 8]. Based on the data from 113 countries, Surkalim et al. [9] concluded that loneliness is occurring at problematic levels globally, with the highest rates of loneliness consistently found in Eastern European countries, which include the Czech Republic. A higher risk of loneliness has been linked to the female sex, low socioeconomic status, young and older people and chronic medical conditions [10].

Loneliness has a significant impact on both physical and mental health. So far, it has been associated with depression and anxiety [11], psychotic symptoms [12], cognitive impairment [13], paranoia [14] and even suicidal ideation and behaviors [15]. Regarding physical health, lonely people are more likely to develop coronary heart disease and stroke [16], Alzheimer’s disease [17] and have a higher risk of premature mortality, hospitalization and an emergency department visit [18]. Overall, the mortality risk connected with loneliness and social isolation is similar to obesity, substance use, mental health problems or a lack of access to healthcare [19].

As a painful experience, loneliness can lead to unhealthy behaviors, such as alcohol and drug use [20, 21], smoking, caffeine intake [22] or problematic Internet use [23]. However, some studies have come to different conclusions. For example, the study of [24] found a positive association for non-cannabis drugs only. Similarly, the relationship between the frequency of alcohol consumption and loneliness remains ambiguous, as studies have reported mixed results: loneliness was connected to more frequent or risky alcohol use by [25] or [26], whereas [11] and [27] reported a negative link between these variables. Moreover, some studies, e.g., [28] or [29], found no significant association between these variables. Recent systematic reviews have shown that considerably less research has been paid to the relationship between loneliness and smoking [30] and loneliness and coffee intake [31]. A study by [32] concluded that smokers felt lonelier than non-smokers, whereas [33] yielded the opposite result. A systematic review by Dyal and Valente [30] reported that merely half of the studies conducted found any association between loneliness and smoking, with a negative association in only one of the studies. Regarding the impact of social isolation due to the COVID-19 lockdown on changes in caffeine consumption, results show that for most individuals, caffeine consumption has not changed [31].

There is growing evidence that loneliness is associated with problematic Internet use (PIU), defined as excessive or poorly controlled urges or behavior connected to Internet use, leading to distress and disrupting major areas of life [34]. These associations have been found in various countries in Europe, Asia, and the United States [23, 35]. According to a recent meta-analytic review [36], the relationships between PIU and loneliness were stronger in studies from South Asia and Europe. However, based on current reviews [35, 37], the majority of these studies were focused on adolescents and young adults [38–40] and there is a lack of research targeting middle-aged and older adults, e.g., [41, 42]. Therefore, the aim of this study is to identify sociodemographic determinants of loneliness and to investigate the relationship between loneliness and frequency of substance use and PIU in different age groups in Czech society.

## Methods

### Participants and Procedure

We designed an online questionnaire to gather the necessary data. This survey was distributed by a professional agency (The Czech National Panel, Prague, Czech Republic) through its network of regular respondents. To achieve a balanced sample close to national characteristics regarding age and gender, the respondents were selected randomly using socio-demographic quotas of the Czech population. The data were obtained on the Czech adult population in April 2021 during a partial COVID-19 lockdown, when social distancing measures were in place [43]. Given the online nature of the questionnaire, to ensure high data quality, the following exclusion criteria were introduced: 1. completing the survey too quickly (under 15 min for a survey which normally lasted about 45 min); 2. inconsistent responses for repeated questions on age, height and weight (i.e., a difference of two or more units of the measure); 3. missing answers on our variables of interest). In total, 1,662 participants finished the survey, and 369 were excluded due to the criteria mentioned above. Therefore, the final sample comprised 1,293 participants aged 18–92 years (mean age = 50.1 years, SD = 15.4; 56% male).

At the beginning of the survey, participants were informed in writing about the purpose of the study, anonymization and the confidential treatment of the data. Participation in the research was fully voluntary. Respondents were required to express explicit agreement with each key point of the informed consent before starting the survey. While filling in the survey, participants could continue to the next question only after finishing the current one. However, they had the option to withdraw at any time without giving a reason. Prior to the main study, a pilot study was conducted among volunteers at Palacky University Olomouc. The study design was approved by the Ethics Committee of Palacky University Olomouc (No. 2020/06).

### Measures

#### Loneliness

The Three-Item Loneliness Scale (TILS) [4] was used to measure perceived *loneliness*. It was designed for large surveys by selecting items from the UCLA Loneliness Scale [44]. The responses are coded on a three-point Likert scale: *1 = Hardly ever*, *2 = Some of the time*, *3 = Often.* Individual scores were summed up, with the total score ranging from 3 to 9. A higher score corresponds to greater feelings of loneliness. For statistical analyses and the graphical representation, the summary score of loneliness was categorized according to [45]: *none* (3), *mild* (4–5), *moderate* (6–7) and *severe* (8–9). In the present study, the reliability of the scale was satisfactory, with Cronbach’s alpha = 0.72.

#### Substance Use

To assess the *frequency of drug use, alcohol use, smoking and caffeine consumption,* participants were asked the questions: “How often in the past month have you*:* a) *used illegal drugs?* b) *drunk alcohol?* c) *smoked?* d) *drunk coffee?*” with response possibilities: *1 = Never*, *2 = About once or twice*, *3 = About every week*, *4 = More than once a week*, *5 = Everyday*, *6 = Many times a day*.

To capture the tendency of individuals to use addictive substances, we introduced the *All-substance use* score, which was calculated by summing the scores for each substance, such as drugs, alcohol, smoking and caffeine. We are aware that the health consequences of frequent use of caffeine, cigarettes, alcohol and drugs are not identical. However, we included caffeine in the study because it has negative health consequences when exceeding a daily dose that is equivalent to 400 mg/day for healthy adults and is significantly lower for groups with health limitations [46]and because of its addictive character, which produces behavioral and physiological effects similar to other addictive substances [47]. Thus, in our study, we reasoned similarly as Bruno et al. [22], who added a point for each increase in consumption of alcohol, cigarettes, coffee, hypnotics, and comfort foods on a scale characterizing unhealthy lifestyle change. Scores ranged from 4 to 24, with higher scores corresponding to more frequent substance use. Since the substance use scale is not a homogeneous scale, Cronbach’s alpha was not reported.

#### Problematic Internet Use

The General Problematic Internet Use Scale-2 (GPIUS-2) [48] was used to measure *PIU*. The GPIUS-2 consists of 15 items divided into five subscales: Preference for Online Social Interaction, Mood Regulation, Cognitive Preoccupation, Compulsive Internet Use and Negative Outcomes. Items were rated on a seven-point Likert scale ranging from *1 = Definitely disagree* to *7 = Definitely agree.* The total score (ranging from 15 to 105) was obtained by adding up all items, with higher scores indicating greater severity of PIU. Although the GPIUS2 scale has been adapted and validated in adult populations, e.g., in the United States [48], Portugal [49], the older population has been underrepresented. Caplan [48] notes that studies on PIU include individuals who are likely to use the Internet frequently. Therefore, caution is needed in generalising the results, especially to older populations where frequent Internet use may not be as prevalent. In the present study, the reliability of the scale was high, with Cronbach’s alpha = 0.92.

Sociodemographic characteristics, such as gender (male-female), age, family status, employment status and education level, were obtained by the questionnaire. The questionnaires used can be found in Supplementary File S1.

### Statistical Analysis

In the first step, we described the sociodemographic characteristics of the study sample and perceived loneliness in each group. To compare the differences in loneliness among the sociodemographic groups, we used the t-test for gender and one-way ANOVA for the rest of the variables with more than two categories. To evaluate the relationship between loneliness and substance use, non-parametric Spearman’s correlation coefficients were used for each age group, since loneliness was coded as an ordinal variable. Next, the effect of loneliness and sociodemographic groups (predictors) on substance use and PIU (dependent variables) were assessed with multivariate linear regression models. In order to assess the interaction effect of loneliness and age in the final regression models, the two predictor variables were employed in their original continuous version. All analyses were performed using the statistical software IBM SPSS version 21 (IBM Corp., Armonk, NY, United States).

## Results

Table 1 describes the sociodemographic characteristics of the study sample. Of all the respondents, 229 (17.7%) reported that they were not lonely, 497 (38.4%) reported mild loneliness, 471 (36.4%) reported moderate loneliness and 96 (7.4%) reported severe loneliness. There were significant differences (*p* < 0.001) in the level of loneliness found between the sociodemographic groups in gender, age, marital status, employment status, as well as education level. The highest levels of perceived loneliness were associated with young people, female gender, having no partner, no paid employment and elementary education. The levels of PIU between the sociodemographic groups were differed significantly (*p* < 0.05) in age, employment status and education level. The highest PIU rates were associated with young age, no paid employment and elementary education. In terms of all-substance use, significant differences were found between sociodemographic groups in age and education, with the highest values for the late-middle age and secondary vocational education level.

**TABLE 1:** Descriptive characteristics of the sample (Czechia, 2021).

Sociodemographic group	*N*	%	Loneliness	PIU^a^	All-substance use
			M (SD) *p-value*	M (SD) *p-value*	M (SD) *p-value*
Total	1,293	100	5.16 (1.53)	32.98 (12.93)	10.26 (3.36)
Gender			<0.001	0.284	0.810
Male	730	56.5	5.00 (1.49)	32.64 (13.01)	10.24 (3.38)
Female	563	43.5	5.36 (1.56)	33.42 (12.82)	10.28 (3.10)
Age			<0.001	<0.001	<0.001
Young adulthood (18–34 years)	242	18.7	5.44 (1.53)	36.62 (12.45)	9.57 (3.45)
Early middle age (35–49 years)	446	34.5	5.22 (1.49)	33.87 (12.84)	10.42 (3.44)
Late middle age (50–65 years)	320	24.8	4.90 (1.60)	31.56 (12.98)	10.81 (3.13)
Elderly (66–92 years)	285	22.0	5.13 (1.47)	30.10 (12.58)	9.97 (2.80)
Family status			<0.001	0.660	0.900
Married/partnership	829	64.1	5.00 (1.47)	32.49 (13.03)	10.26 (3.16)
Single/divorced/widow(er)	464	35.9	5.44 (1.60)	33.87 (12.72)	10.24 (3.44)
Employment status			< 0.001	< 0.001	0.480
With a paid job^b^	737	57.0	5.02 (1.50)	33.36 (12.72)	10.32 (3.37)
Without a paid job^c^	152	11.8	5.73 (1.54)	37.09 (13.52)	9.97 (3.62)
Disabled/old-age pensioner	404	31.2	5.20 (1.53)	30.75 (12.67)	10.25 (2.90)
Education level			<0.001	0.010	<0.001
Elementary	79	6.1	5.77 (1.60)	37.03 (14.01)	10.19 (3.81)
Secondary vocational	482	37.3	5.23 (1.48)	33.43 (12.90)	10.67 (3.35)
Secondary graduation	401	31.0	5.06 (1.55)	31.89 (12.63)	10.39 (3.12)
College/University^d^	331	25.6	5.03 (1.53)	32.69 (12.92)	9.51 (3.03)

^a^ Notes: Problematic Internet use.

^b^ Including employed, self-employed, entrepreneur, part-time job.

^c^ Including student, household, without work, maternity leave.

^d^ Including higher vocational school.

The associations between loneliness and substance use are shown in Table 2. No significant relationship was found between loneliness and drugs, alcohol, smoking or caffeine consumption. The analysis revealed a significant positive correlation between loneliness and PIU for all age groups, with a medium effect size for young adults and a small effect size for other age groups. The age groups differed the most in all-substance use, with only the young group having a significant positive relationship, with a small effect size.

**TABLE 2:** Spearman’s correlation coefficients between loneliness and substance use, and loneliness and problematic Internet use stratified by the age groups (Czechia, 2021).

Age group	Correlation of loneliness & drugs, loneliness & alcohol, loneliness & smoking, etc.					
	Drugs	Alcohol	Smoking	Caffeine	All-substance use	PIU^a^
All	0.051	−0.002	0.003	0.001	0.0005	0.199***
Young	0.019	0.060	0.111	0.083	0.140*	0.258***
Early middle	0.056	−0.004	−0.003	−0.008	−0.010	0.172***
Late middle	0.092	−0.048	−0.047	0.052	−0.028	0.188***
Elderly	−0.038	0.0004	0.003	0.030	0.002	0.139*

Notes: *p < 0.05, **p < 0.01, ***p < 0.001.

^a^ Problematic Internet use.

The effect of loneliness and the sociodemographic variables on PIU and all-substance use is presented in Supplementary Files S2, S3. The only significant predictors of PIU were loneliness and age. The sole significant predictor of all-substance use was age. Therefore, only loneliness and age were considered for further analyses.

Descriptive statistics (Table 3) showed that the severity of PIU increased with increasing levels of loneliness. There was a significant difference between loneliness groups in PIU. *Post-hoc* comparisons show the largest difference between the non–severe loneliness group, with a large effect size (MD = −8.562, p_bonf_ < 0.001; d = 0.685). There was no significant difference between loneliness groups in all-substance use.

**TABLE 3:** Descriptive table of problematic internet use and all-substance use, stratified by loneliness groups and age groups (Czechia, 2021).

Group	PIU^a^			All-substance use		
	M	SD	*p*-value	M	SD	*p*-value
Loneliness			<0.001			0.604
None	29.80	13.25		10.32	3.60	
Mild	31.28	12.44		10.25	3.19	
Moderate	35.26	12.19		10.15	3.25	
Severe	38.20	14.73		10.65	2.80	
Age group			<0.001			0.031
Young adulthood (18–34 years)	36.62	12.45		9.57	3.45	
Early middle age (35–49 years)	33.87	12.84		10.42	3.44	
Late middle age (50–65 years)	31.56	12.98		10.81	3.13	
Elderly (66–92 years)	30.10	12.58		9.97	2.80	

^a^ Notes: Problematic Internet use.

Descriptive statistics (Table 3) showed that PIU decreased with age regardless of loneliness, with the highest level found in the young group and the lowest in the elderly group. According to the *post hoc* analysis, the largest difference was found between the young and the elderly group, with a medium effect size (MD = −5.306, p_bonf_ < 0.001, d = 0.424). Regarding all-substance use, the highest frequency was found in the late middle-aged and the lowest in the young group. The *post hoc* analysis revealed no significant difference between age groups in all-substance use.

To explore the effect of loneliness and age together with their interaction on PIU and all-substance use, further multivariate linear regression models were employed (see Table 4). Loneliness was positively associated with PIU, i.e., higher level of loneliness corresponded to higher level of PIU. Age was not a significant predictor of PIU, neither was the interaction of age and loneliness. In case of all-substance use, age was a positive significant predictor while loneliness and the interaction term were not significant at the confidence level of 95%.

**TABLE 4:** Results of multivariate linear regression models assessing the effect of loneliness and age (predictors) on PIU and all-substance use (dependent variables) (Czechia, 2021).

Predictors	Beta coefficient^b^	Standard error	t	*p*-value
Outcome: PIU^a^				
Loneliness	0.232	0.765	2.562	0.011
Age	−0.131	0.081	−1.347	0.178
Loneliness × Age	−0.054	0.015	−0.435	0.664
Outcome: All-substance use				
Loneliness	0.179	0.200	1.909	0.056
Age	0.226	0.021	2.238	0.025
Loneliness × Age	−0.241	0.004	−1.861	0.063

^a^ Notes: Problematic Internet use.

^b^ Standardized.

**FIGURE 1:**
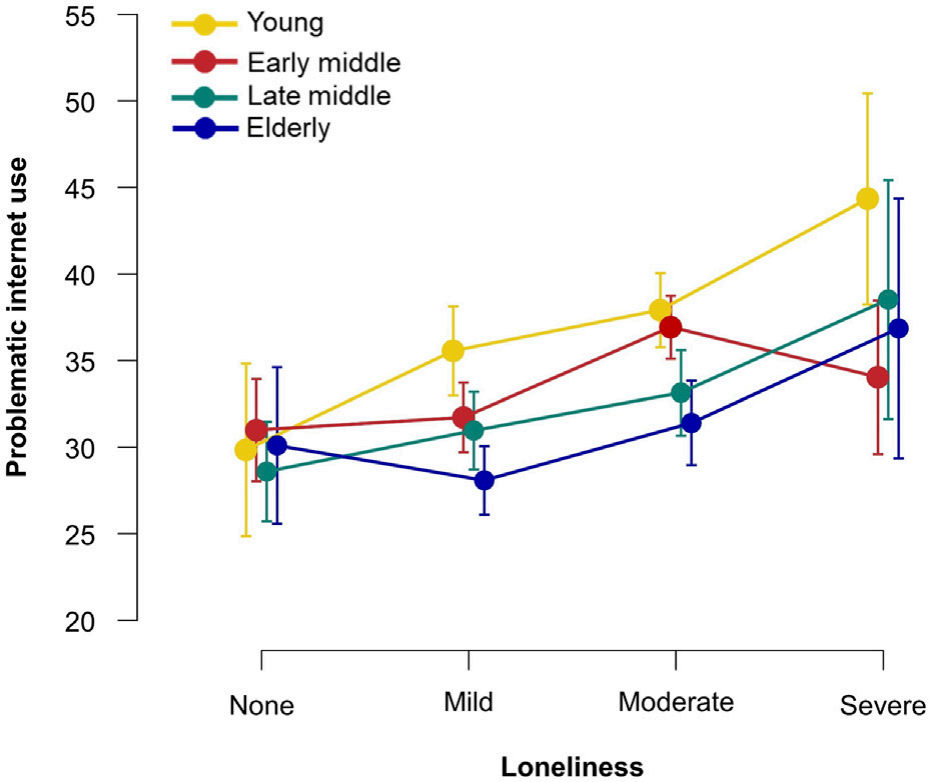
Loneliness and problematic internet use across age groups, group means and 95% confidence interval values are depicted (Czechia, 2021).

**FIGURE 2:**
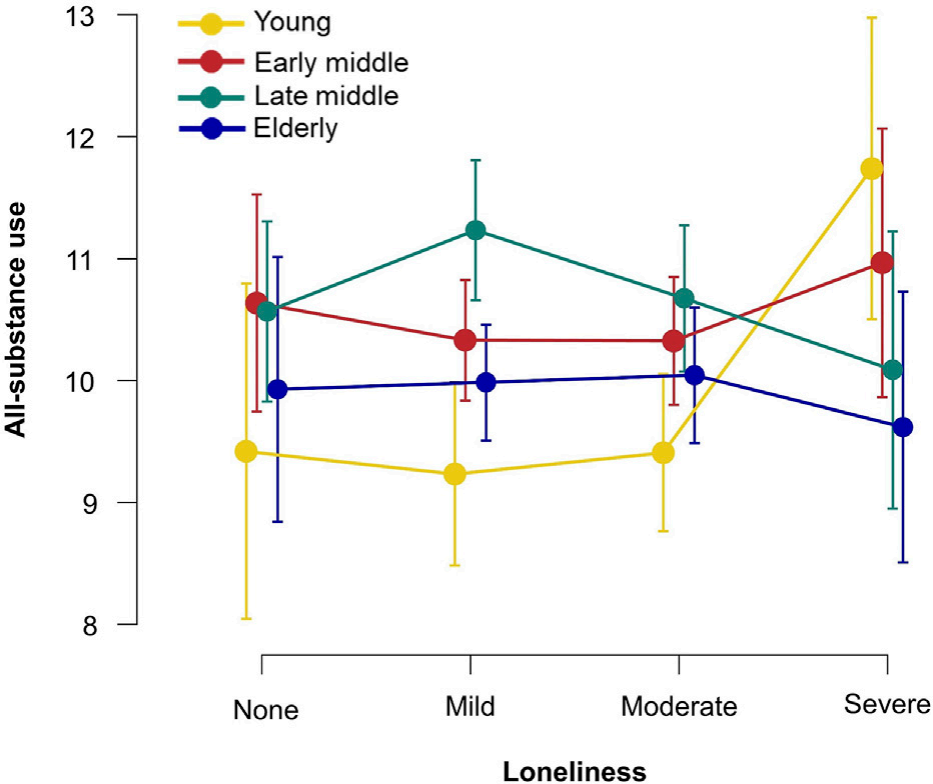
Loneliness and all-substance use across the age groups, group means, and 95% confidence interval values are depicted (Czechia, 2021).

To better understand the relationship between loneliness, PIU and all-substance according to age, group means were graphically compared (see Figures 1, 2). Figure 1 illustrates the association between loneliness and PIU stratified by age groups. It suggests that the young and late middle-age groups show an increasing trend, i.e., the level of PIU increases with higher loneliness. In the young group, however, the line segments show greater slopes, and the PIU score is higher for all loneliness levels. Therefore, the young group seems more affected by loneliness than the late middle-age group. The curve for the early middle-age group shows an increase in PIU connected to a moderate level of loneliness, followed by a decrease in PIU in the severe loneliness group. The curve for the elderly is U-shaped, with the highest PIU values in the severe loneliness group. In the mild, moderate and severe levels of loneliness, the young group scores the highest on the PIU scale compared to the other age groups.

Figure 2 shows the associations between loneliness and all-substance use, stratified by age groups. There is no clear downward or upward trend for any age group. For the young group, there is a large difference in all-substance use at the severe level of loneliness compared to lower levels. The figure shows that the early middle-age and elderly groups are not greatly affected by loneliness, as their curve does not differ by more than one point in the all-substance use scale. The curve for late middle-age has an inverted U-shaped, with the highest values in the mild loneliness group and the lowest in the severe loneliness group.

## Discussion

This study aimed to assess the associations between loneliness and the frequency of substance use—such as alcohol and drug use, caffeine intake, smoking—and PIU in different age groups. Nearly half of the respondents reported moderate to severe loneliness. The highest levels of perceived loneliness were associated with young people, female gender, having no partner, no paid employment and with elementary education. The results showed no significant effect of loneliness on all-substance use. Specifically, there was no significant relationship between loneliness and the frequency of alcohol or drug use, caffeine intake or smoking. Loneliness affected the rates of PIU for each age group, with higher loneliness corresponding to higher PIU.

In our study, nearly half of the respondents reported moderate to severe levels of loneliness during a partial COVID-19 lockdown. Compared to data measured prior to the COVID-19 pandemic [50], there has been a noticeable increase in loneliness of 20% in the Czech Republic. According to a systematic review [5], there was a small increase in loneliness during COVID-19 in most countries studied in Europe and the United States. However, the results are heterogeneous due to the time and intensity of the restrictions, the population studied and the instruments used to measure loneliness. The significant increase in loneliness in the Czech Republic at the time of the COVID-19 pandemic may have been due to strict restrictions that lasted intermittently for about 7 months [43] and were loosened only at the time of our data collection.

In line with previous studies [10, 11], higher loneliness in the Czech Republic was associated with female gender, low socioeconomic status and not having a partner. However, women’s more frequent feelings of loneliness may be explained by the fact that men are more reluctant than women to express negative feelings such as loneliness [51]. This is consistent with our results, though using an online questionnaire may, according to [52], provide a safer environment for respondents to express negative feelings. Women also tend to live longer than men, so they are more likely to experience the loss of a spouse, which can be a significant factor in loneliness [52].

In line with Hansen and Slagsvold [50], loneliness was lower in groups with higher education, which may be due to their better social competence and higher economic status. In terms of age, the research to date is not entirely consistent; for example, a meta-analysis by [9] found the highest rates of loneliness in older age groups, whereas a meta-synthesis by [10] found the highest loneliness in the young and older people. Our results are in agreement with [10], where the relationship between age and loneliness was U-shaped, with high values for young and elderly compared to middle age. A likely explanation is that middle-aged people tend to be more socially involved in work and family life than the younger and older age groups.

The results did not show an effect of loneliness on substance use. Our results are consistent with [28] and [29], who found no relationship between loneliness and alcohol consumption, and with [31], who found no change in coffee consumption during the COVID-19 lockdown for most people. In contrast to our findings, a study by [22] concluded that social isolation leads to a tendency towards unhealthy habits, which included the increased frequency of alcohol, tobacco and coffee use. A possible explanation for the different results is offered by [24], who found different results between within-person and between-person effects of loneliness on alcohol, implying that loneliness increases solitary consumption but decreases social consumption. As with alcohol, we can assume that drug use, smoking and coffee drinking may represent solitary consumption or, conversely, a means of social interaction.

Our study showed the effect of loneliness on problematic Internet use, with PIU increasing with higher levels of loneliness, which is consistent with previous literature [35]. Based on this finding, we hypothesize that nowadays, the Internet represents a quicker and easier way for most people to cope with the feeling of loneliness than substance use. Internet use itself can exacerbate or reduce loneliness [37] and represents a valuable tool for deepening relationships and establishing new ones [53]. However, PIU leads to increased loneliness and, according to our results, represents a means of escape from the social world rather than a way to connect with others.

Given the lack of research examining the role of age in the relationship between PIU and loneliness, Moretta and Buodo [37] suggest exploring it more closely. In our data, no interaction effect of loneliness and age group was found for PIU. We discovered that PIU rates increased with increasing loneliness regardless of age; however, age groups differ in the strength of this relationship. Therefore, PIU appears to be a more common maladaptive method of coping with loneliness than substance use in every age group.

### Strengths and Limitations

The strength of the study was the use of a large sample close to the characteristics of a nationally representative sample regarding age and gender distribution. To the best of our knowledge, this study provides the most up-to-date information on loneliness in one of the countries with the highest loneliness rates, which is the Czech Republic [9]. It is also the first to use the TILS scale in the Czech society. A limitation of our data is that the continuous variables used in the analyses do not meet the assumption of normality. Nevertheless, violation of normality of the error terms should not bias the results, as the linear regression is a robust method by the law of large numbers and the central limit theorem [54]. Further, we have used self-rating scales that may be biased by social desirability and impression management. These are particularly sensitive issues related to substance use, where people may tend not to admit the frequency of substance use. Given the online environment and the assurance of anonymity, bias could be reduced. The data were collected during the COVID-19 pandemic, which may bias the results, especially in terms of the observed prevalence of loneliness. The last limitation is the use of a cross-sectional design from which causal inferences cannot be drawn.

### Implications

Our findings highlight the large degree of loneliness experienced by almost half of the Czech population in the context of the COVID-19 pandemic. Higher rates of loneliness were linked to higher rates of PIU. Therefore, professional help for lonely people should focus not only on substance use but turn its attention to PIU in all age groups. Professionals can help people find ways to use the Internet to build and deepen relationships. This may include helping clients in finding friends who share their interests, communicate online with those close to them and look for interest groups and leisure time activities to join.

For future research, we recommend distinguishing between social and lonely substance use [55], mainly smoking, coffee and alcohol drinking, which could offer deeper insight into this area. Future research could also provide information on the current state of loneliness after the end of the COVID-19 pandemic and causal inferences to confirm our claims.

### Conclusion

Our results show that almost half of the people in the Czech Republic experienced problematic levels of loneliness during the COVID-19 pandemic. Higher loneliness was associated with greater severity of PIU. This trend occurs regardless of age. The result adds further evidence for addressing the problem of loneliness, which is becoming a health policy priority in the 21st century.
