# Supplementary material for: Loneliness Is Associated With Problematic Internet Use but Not With the Frequency of Substance Use: A Czech Cross-Sectional Study
Source: Int J Public Health. 2023 Nov 2;68:1606537. doi: 10.3389/ijph.2023.1606537 (PMC10651728; doi:10.3389/ijph.2023.1606537)
Supplement: Supplementary file 1 [file DataSheet2.pdf]

Supplementary File 2: *Results of a multivariate linear regression model assessing the effect of loneliness and sociodemographic groups (predictors) on PIU (dependent variable) (Czech Republic, 2021).*

Predictors	Beta coefficient	Standard Error	t	p-value
**Outcome: PIU[1]**				
Loneliness				
None	reference			
Mild	1.303	1.011	1.289	0.198
Moderate	4.744	1.030	4.606	< .001
Severe	7.584	1.554	4.880	< .001
Gender				
Male	reference			
Female	0.026	0.732	0.036	0.972
Age group				
Young adulthood (18–34 yrs)	reference			
Early middle age (35–49 yrs)	-2.115	1.047	-2.021	0.044
Late middle age (50–65 yrs)	-3.719	1.182	-3.148	0.002
Elderly (66–92 yrs)	-4.530	1.586	-2.856	0.004
Family status				
Married/ partnership	reference			
Single /divorced/ widow(er)	0.668	0.738	0.905	0.366
Employment status				
With a paid job[2]	reference			
Without a paid job[3]	1.430	1.243	1.151	0.250
Disabled/old-age pensioner	-0.973	1.223	-0.795	0.427
Education level				
Elementary	reference			
Secondary vocational	-1.315	1.587	-0.828	0.408
Secondary graduation	-2.103	1.632	-1.289	0.198
College/University[4]	-2.119	1.663	-1.275	0.203

Notes: [1]problematic Internet use, [2]including employed, self-employed, entrepreneur, part-time job; [3]including student, household, without work, maternity leave; [4]including higher vocational school
