# Supplementary material for: Loneliness Is Associated With Problematic Internet Use but Not With the Frequency of Substance Use: A Czech Cross-Sectional Study
Source: Int J Public Health. 2023 Nov 2;68:1606537. doi: 10.3389/ijph.2023.1606537 (PMC10651728; doi:10.3389/ijph.2023.1606537)
Supplement: Supplementary file 2 [file DataSheet3.pdf]

Supplementary File 3: *Results of a multivariate linear regression model assessing the effect of loneliness and sociodemographic groups (predictors) on all-substance use (dependent variable) (Czech Republic, 2021).*

Predictors	Beta coefficient	Standard Error	t	p-value
**Outcome: All-substance use[1]**				
Loneliness				
None	reference			
Mild	-0.005	0.260	-0.019	0.985
Moderate	-0.092	0.265	-0.349	0.727
Severe	0.452	0.399	1.132	0.258
Gender				
Male	reference			
Female	-0.088	0.188	-0.467	0.640
Age group				
Young adulthood (18–34 yrs)	reference			
Early middle age (35–49 yrs)	0.721	0.269	2.680	0.007
Late middle age (50–65 yrs)	0.994	0.304	3.273	0.001
Elderly (66–92 yrs)	0.297	0.408	0.729	0.466
Family status				
Married/ partnership	reference			
Single /divorced/ widow(er)	-0.080	0.190	-0.424	0.672
Employment status				
With a paid job[2]	reference			
Without a paid job[3]	-0.229	0.319	-0.718	0.473
Disabled/old-age pensioner	-0.045	0.314	-0.143	0.886
Education level				
Elementary	reference			
Secondary vocational	0.209	0.408	0.511	0.609
Secondary graduation	-0.051	0.419	-0.121	0.904
College/University[4]	-0.834	0.427	-1.952	0.051

Notes: [1]Drugs, alcohol, smoking, caffeine consumption, [2]including employed, self-employed, entrepreneur, part-time job; [3]including student, household, without work, maternity leave; [4]including higher vocational school
