# Supplementary material for: Loneliness Is Associated With Problematic Internet Use but Not With the Frequency of Substance Use: A Czech Cross-Sectional Study
Source: Int J Public Health. 2023 Nov 2;68:1606537. doi: 10.3389/ijph.2023.1606537 (PMC10651728; doi:10.3389/ijph.2023.1606537)
Supplement: Supplementary file 3 [file DataSheet1.pdf]

Supplementary file 1

THE THREE-ITEM LONELINESS SCALE (TILS)

Citation Information:

Hughes ME, Waite LJ, Hawkley LC, Cacioppo JT. A Short Scale for Measuring Loneliness in Large Surveys. *Res Aging*. 2004;26(6):655–72

The next questions are about how you feel about different aspects of your life. For each one, tell me how often you feel that way.

1 Hardly ever
2 Some of the time
3 Often

1. First, how often do you feel that you lack companionship?
2. How often do you feel left out?
3. How often do you feel isolated from others?

Range from 3 to 9.

SUBSTANCE USE

How often in the past month have you:

1. used illegal drugs?
2. drunk alcohol?
3. smoked?
4. drunk coffee?

1 Never
2 About once or twice
3 About every week
4 More than once a week
5 Everyday
6 Many times a day

All-substance use score = sum of the score for drugs, alcohol, smoking and caffeine. Range from 4 to 24.

GENERALIZED PROBLEMATIC INTERNET USE SCALE 2 (GPIUS 2)

Citation Information:

Caplan, S. E. (2010). Theory and measurement of generalized problematic Internet use: A two-step approach. *Computers in Human Behavior, 26,* 1089–1097.

Instructions: Please use the scale below to rate the extent to which you agree with each of the following statements:

1 Definitely disagree
2 Disagree
3 Slightly disagree
4 Neither agree nor disagree
5 Slightly agree
6 Agree
7 Definitely agree

1 I prefer online social interaction over face-to-face communication.
2 I have used the Internet to talk with others when I was feeling isolated.
3 When I haven't been online for some time, I become preoccupied with the thought of going online
4 I have difficulty controlling the amount of time I spend online.
5 My internet use has made it difficult for me to manage my life.
6 Online social interaction is more comfortable for me than face-to-face interaction.
7 I have used the Internet to make myself feel better when I was down.
8 I would feel lost if I was unable to go online.
9 I find it difficult to control my Internet use.
10 I have missed social engagements or activities because of my Internet use.
11 I prefer communicating with people online rather than face-to-face.
12 I have used the Internet to make myself feel better when I've felt upset.
13 I think obsessively about going online when I am offline.
14 When offline, I have a hard time trying to resist the urge to go online.
15 My Internet use has created problems for me in my life.

Range from 15 to 105.

Item for each subscale (either sum or average the items listed to obtain a score for the subscale).

Preference for Online Social Interaction: 1, 6, 11
Mood Regulation: 2, 7, 12
Cognitive Preoccupation: 3, 8, 13
Compulsive Internet Use: 4, 9, 14
Negative Outcomes: 5, 10, 15
